# The Treatment of Epilepsy

**Published:** 1910-07-02

**Authors:** W. Aldren Turner

**Affiliations:** Physician to the Hospital, and Physician to King's College Hospital.


### Hospital Clinics.

### THE TREATMENT OF EPILEPSY.

By W. ALDREN TURNER, M.D., F.R.C.P.; Physician to the Hospital, and Physician to King's College Hospital.

(A Lecture delivered at the National Hospital for the Paralysed and Epileptic, Queen Square, W.C., on Friday, June 10, 1910.)

Gentlemen,—My lecture to-day is on the treatment of epilepsy. I want you to understand that the term is applied in relation to the idiopathic disease—that is, to a disease which usually arises about the time of puberty, approximately from thirteen to fifteen years of age, generally without any ascertainable or tangible cause. These persons have usually a hereditary history, either to epilepsy or to some form of nerve disease or degeneration. If you limit the term idiopathic epilepsy to a case of fits having such hereditary history and such origin, you will include most of the cases of ordinary epilepsy which come before you. I do not want to say anything about the treatment of organic epilepsy.

### Petit Mal Moteur.

It is now necessary to mention what is the character of the fit or discharge which should be included under the term epilepsy. To my mind, all that you require to prove is impairment or loss of consciousness. If you can establish that a person has had temporary sudden loss or impairment of consciousness, you have established the disease epilepsy: it is not essential to show convulsion, psychical changes other than impairment of consciousness, involuntary evacuation of the bladder, or biting of the tongue. I think that is a very important matter, because the definition of epilepsy sometimes arises in Courts of Law; and you might on any occasion, in the witness-box, he asked what you mean by epilepsy. If you accept that as a complete definition of epilepsy, you can eliminate a large number of epileptoid, or epileptiform phenomena. There is a common type of epilepsy called *petit mal moteur*, characterised by "jerks" or "jumps." Sometimes it constitutes the whole character of the disease in any particular case. These jumps may not be associated in every instance with impaired consciousness; but, if you probe sufficiently deeply, you will find that a person who is suffering from these attacks of *petit mal moteur* has had at some time loss or impairment of consciousness. Those who attend the out-patients will every now and again see cases of psychical epilepsy, which may also be included in our definition. It also includes aura sensations, or abortive attacks in which the whole seizure is the aura of the major fit. For example, a person may complain of a sensation in the stomach which passes upwards towards the head; then consciousness is abolished, and an attack ensues. These patients may have simple attacks of epigastric sensation which ascends to the head. It is known as an aura or warning fit; and you may include under the term epilepsy those auræ sensations or warning fits which are not uncommon in those who suffer from major epilepsy. And under the definition you can include convulsive fits, whether of the minor or the major type. You can also include an interesting type of epilepsy—namely, so-called psychical epileptic equivalents—in other words, attacks of mental disturbance, sometimes merely automatism, which are said to replace the fits.

### Notes on Treatment.

Let me now say a few words as to how best to treat the disease, because you must be prepared with a number of variations in your treatment. Of all the diseases which we have to treat, epilepsy is the one which most taxes our therapeutic and other resources.

First of all, with regard to the drug treatment, there is one series of drugs which, though not actually specific, comes near it, and that is the salts of bromine. But the salts of bromine, as most of you who have treated many epileptics know, are very uncertain in their action. You may treat a patient for months on bromide, varying the dose and giving the various salts, and yet you will obtain most unsatisfactory results. So the first point is that you must be prepared for great disappointment in the treatment of a large number of cases of epilepsy by means of bromine salts. About 50 per cent. of your cases of epilepsy will not respond to bromides, and if they do not, the chances are that they will respond to nothing else. What can you do for the other 50 per cent. in which the treatment is of some use? When you see epilepsy at its commencement, or in the first year or two, you should prescribe bromide salts for a time. If they are going to respond favourably, they will do so rapidly. Quite a number of cases are arrested by this means—arrested for years, sometimes permanently—so that we come to regard some cases of epilepsy as eminently curable. What are the features of these curable cases of epilepsy? First, the presence of a definite hereditary history. Secondly, fits, the *grand mal* type of seizure. Thirdly, fits occurring at long intervals. Fourthly, the absence of marked mental impairment. If you can establish these features in a case, and there are many in which you can, you are dealing with one in which it is well worth while persevering with the treatment. Most of the cases, however, do not show such favourable phenomena—the fits occurring much more frequently, consisting of both major and minor attacks, and having often a psychical element in them. How should you treat these cases? You start with your bromides, and you may or may not get a good result at the outset. If you fail to influence the case by one form of bromide salt, try another. The salt I use most, and which I think best, is the bromide of sodium, 20 to 60 grains in the 24 hours. Some people prefer the potassium salt, others a combination of bromides, so that not more than 60 grains are given in 24 hours. In this latter case, should you divide up your doses, and give, say, two large doses in the 24 hours? That depends very much on the frequency of the seizures. If it is a nocturnal case, I think the best plan is to give the bromide at bedtime, 30 to 40 grains, well diluted with water. If you do not get from this the effect which you think you ought to get, you may in addition alter the patient's diet.

### Dietetics.

I think this is a very good place to make one or two remarks about the dietetic treatment of epilepsy, because I think you get better results in your treatment of epilepsy if you pay attention to the diet; in fact, you can then reduce your dose of bromide more satisfactorily. The first point is the elimination of salt from the food. That has only quite recently come into anything like general use. The theory dominating this restriction is the following: one of the normal constituents of the blood serum is sodium chloride. If you can replace the chloride element of the blood by bromide, you are doing something which is favourable to the arrest of the seizures. It has been found that one-third of the chlorine of the blood serum has to be replaced by an equivalent amount of bromine. When more than this occurs, bromide intoxication results. When less chlorine is ingested, saturation takes place sooner. When the patient takes salt-free diet, saturation takes place in three to four days. The practical application of that is, if you take away the salt from the diet of epileptics, a smaller dose of bromide need be given to cause saturation: a 60-grain dose can be reduced to 40. Some observers think this increases the danger of bromism, but, though that may be so in some cases, I have not seen it myself. My experience of the method has been most satisfactory, and I recommend it to be tried in all cases, at all events at the commencement. The second point is the elimination of the purin element of the food. There has been much speculation lately as to whether purins play a part in the production of toxic symptoms. The matter requires fuller investigation than it has yet received; but, so far, I have had satisfactory results from attention to this point. The foods which contain purins are all forms of meat, poultry, game, some forms of fish—turbot, halibut, salmon—and peas, beans, lentils. Foods which may be regarded as purin-free are eggs, milk, cream, cheese, butter, olive oil, practically all the vegetables in common use, fruits (dry and fresh), honey, rice, tapioca, macaroni.

### Combined Drugs.

But there are a number of cases in which you will find that the administration of some other drug in combination with the bromide may be of considerable assistance. The drugs commonly used in combination with bromides are usually given to combat some features of the case which occur in addition to the seizures: for instance, in patients who have a low arterial tension, or where there is reason to suppose there is a failing action of the heart, or in which there is a valve lesion, you will find the addition of digitalis of great use. Giving digitalis and bromide in combination in minor epilepsy yields satisfactory results. The addition of chloral hydrate to the bromides is often of great use when the fits are beginning to occur in series—what is called serial epilepsy. The bromides may be combined with the glycero-phosphates in cases where there are anæmia and chlorosis, especially in young women whose menstrual functions are not regular. You may combine borax with the bromide; many physicians prefer it to bromide alone. I do not think there is any special advantage in the combination, but there is no doubt that, if a case is not doing well on bromide alone, there may be some use in that combination. I have recently found a combination of bromide, picro-toxine, and the arseniate of antimony useful for cases of epilepsy which have not borne the bromides well.

### Alternatives to the Bromides.

Are there any drugs which you can use instead of the bromides? There is a considerable popular objection to bromides, not only in the lay sense but also among physicians. In many cases bromides have been given in too large quantities. It was considered nothing at one time for a patient to be taking 100 or 150 grains of bromide, with the result that bromism was induced. If you treat cases along the lines I have mentioned, I do not think you will get bad results from bromides. If you can encourage the patients and their friends to continue with them, the results on the whole are satisfactory. But you must be prepared for opposition if you attempt to treat some of your cases with bromides. You may then prescribe other drugs which are of occasional benefit in epilepsy. What are they? First, there is borax (sodium bi-borate). It was introduced by Sir William Gowers at this hospital 20 years ago. Perhaps the best of all non-bromide drugs is belladonna. It was used long before bromides were introduced in the treatment of epilepsy, especially by the French physicians. Belladonna was especially recommended in cases where there was nocturnal incontinence of urine, and it is still of great use in nocturnal epilepsy. You may give 5 or 10m of tincture of belladonna with your bromide, or in the form of a pill. So in all cases of epilepsy in which the bromide salts are not producing the results which you like them to produce you may hopefully prescribe belladonna.

There are some other salts which were used by French physicians a long time ago, and these are the salts of zinc: the oxide, the valerianate, and the lactate. They also fell out of use, but I think they are being resuscitated. A very old remedy—so old that, I suppose, its introduction in the treatment of epilepsy is lost in oblivion—is opium. I think the modern tendency is against using it in epilepsy. A remedy which one would have thought contra-indicated in epilepsy, but which has given some remarkable results, is strychnine, or nux vomica. I do not know how it acts, but probably as a tonic to the vaso-motor centres; and in many cases there is a marked failure of the vaso-motor mechanism. From the little I have seen of them, the salts of calcium seemed to be successful. One has yet to find out what is the type of epilepsy in which salts of calcium are likely to be of service, but the lactate and perhaps the hypophosphite may be well worthy of trial.

Various other forms of treatment have been mentioned, such as giving thyroid gland, but I think the method has disappointed those who have tried it. It is of no use in epilepsy, and I think it makes the fits worse. There is also the injection of blood serum from one epileptic into other epileptics, but that has led to no satisfactory results.

How long are you to continue your treatment of epilepsy? If a case has become a confirmed one, it is not worth while treating it by drugs at all; the case is better looked after in an institution or colony for epileptics. But if after treating a case for a couple of years the fits have ceased, and the patient wants to know whether he is to go on with his medicine, what is your advice? If it is not advisable to stop it after the fits have ceased for a time, how long should you continue it? Many patients take the matter into their own hands. The text-books state that two years' continuance of bromides after the last fit is sufficient. I do not think it is sufficient. We know that remissions of epileptic fits after two years, or even five years, are very common. Even though bromides are continued a relapse will occur. But assuming that after three or five years a relapse has taken place, will you advise the patient to continue taking the medicine? I think he should do so. We have had cases attending here more than 20 years, getting their dose, if not every day, two or three times a week. If at any time they have ceased getting their medicine, they get some return of the sensations, not necessarily of the fits, some aura or suggestion of attack. A person may take 10 grains every night, or three times a week, indefinitely.

### General Treatment.

Now a few remarks about the general or hygienic treatment of epilepsy. Some cases undergo spontaneous cure, and that is not an uncommon type. There is another type, which I referred to as being curable, in which the fits are infrequent and in which there is no mental impairment.

All the organs of the body ought to be systematically examined before you prescribe a remedy. Many cases of epilepsy have certain peripheral irritations which, if relieved, may not actually stop the disease, but may relieve it very much. The nose should be examined for adenoids, or polypi, or any foreign body, the latter especially in children. The eyes should also be examined for errors of refraction. Such errors are common, and however small should be properly corrected. The teeth certainly ought to be examined and put right. Even the careful fitting of a plate to one or both jaws is most useful to epileptics who suffer from their teeth. The stomach and intestines should also be attended to, and the presence of worms in children should be investigated. The question of local disease of pelvic organs in women will arise from time to time. If there is local disease it should, of course, be put right. But do not imagine when a retroverted uterus is put right it will necessarily cure the epilepsy. When you have made these examinations and had the peripheral organs put right, put your patient on the bromide medication on the lines I have mentioned.

### Exercise.

The question may also arise about exercise and about employment in epilepsy. Should a young fellow who has been trained for some profession be put, when it is found he has fits, to market gardening? You should bear in mind that just as there are various types of healthy individuals engaged in various work, so there are different types of epileptics. There is no use in putting a frail boy to strenuous work in a market garden. There are indoor occupations which suit him better. Should these youngsters be encouraged to play cricket, football, tennis, rowing, etc.? I think that an epileptic can do anything in that way as long as it is not accompanied by danger. Bicycling, horse-riding, and swimming should be prohibited.

Then there is the question of sending a patient to an institution for epileptics or treating him at home. On that your opinion will be asked. I think epileptics are better treated at home if their fits are not very frequent. If there are two or three a week, or more, I think such cases are best sent away from home and treated under the care of a doctor in an institution until there is some reduction of fits. Epileptics ought not to be sent to schools with ordinary children; they should be educated privately or at a special school. I think private tuition is best for the epileptic when it can be afforded. When there is marked mental impairment they should be treated and educated as if they were mental defectives.

